# The impact of loneliness on the self-reference effect in remembering and directed forgetting

**DOI:** 10.3389/fpsyg.2026.1809521

**Published:** 2026-06-08

**Authors:** Wanqi Wang, Xiaochen Tang, Yu Wang, Jiaodian Zhou, Chen Hong, Weiman Yan, Ning Chen, Wei Liu

**Affiliations:** 1 School of Psychology, Shanghai Normal University, Shanghai, China; 2 Shanghai Tianlin No. 3 Middle School, Shanghai, China

**Keywords:** cognitive flexibility, directed forgetting, loneliness, memory, self-reference effect

## Abstract

This study investigated the impact of loneliness on the self-reference effect (SRE) under conditions of intentional remembering and directed forgetting. Across two experiments using a self-description paradigm, individuals with high and low levels of loneliness were compared. Results indicated that both groups showed a robust SRE relative to friend- and stranger-referenced items; however, only the high-loneliness group exhibited a mnemonic advantage for negative words. In the directed forgetting task, low-loneliness individuals exhibited greater cognitive flexibility, as they adjusted self-referential memory processing in response to task instructions, whereas high-loneliness individuals exhibited a stable self-referential advantage across both conditions, accompanied by blunted differentiation between friend-referenced and stranger-referenced information. These findings indicate that loneliness is associated with altered self-referential memory processing, characterized by enhanced sensitivity to negative information and reduced flexibility in intentional memory control. By integrating self-reference and directed forgetting paradigms, the present study provides new evidence that loneliness influences not only what individuals remember, but also how flexibly they regulate self-related information in memory.

## 1 Introduction

### 1.1 Loneliness, the self, and cognitive processing

Loneliness refers to the emotional experience arising when an individual’s intrinsic need for intimate connection, companionship, and meaningful social relationships is unmet—that is, when one strongly desires social contact but cannot obtain it (Weiss, 1973). In recent years, loneliness has been increasingly recognized as a widespread and growing public health concern. According to a recent report by the WHO (2025), approximately 16% of the global population experience significant loneliness, with particularly high prevalence among adolescents and young adults. Consistent with these global trends, loneliness is also highly prevalent in China. Existing research suggests that loneliness is common among young adults and university students, with substantial proportions reporting loneliness experiences (Wen et al., 2025). These findings highlight that loneliness is not only common but also represents an important societal and public mental health issue.

Beyond its prevalence, loneliness has profound implications for psychological functioning. Loneliness increases sensitivity to social threat, leading individuals to interpret ambiguous social cues negatively and to engage in cognitive and behavioral patterns that undermine social relationships, thereby reinforcing loneliness in a vicious cycle (Cacioppo and Cacioppo, 2018). Individuals with higher loneliness are more likely to experience social isolation or bullying, adopt maladaptive coping strategies, and face elevated risk of psychological problems (Matthews et al., 2019). For instance, Danneel et al. (2019) found that loneliness and social anxiety mutually reinforce each other: heightened sensitivity to social rejection increases anxiety and avoidance, which reduces actual social support, further exacerbating loneliness. Loneliness also prolongs stress responses, contributing to depressive symptoms (Iob et al., 2020) and to an elevated risk of depression over a 12-year period (Lee et al., 2021). Additionally, loneliness fosters negative expectations toward social interactions, promoting social withdrawal and reinforcing negative affect (Barton et al., 2024). High-loneliness individuals also tend to show poorer self-regulatory abilities, increasing the likelihood of maladaptive behaviors such as problematic internet or smartphone use (Kim, 2018, 2019).

Fundamentally, loneliness is a self-focused psychological experience. The relationship between loneliness and the self may therefore play a central role in understanding how loneliness shapes cognition and behavior. Evidence suggests that loneliness can alter self-concept: reduced interpersonal interaction may induce feelings of fragmentation or identity loss (Motta, 2023). Loneliness also heightens self-focused attention, increasing vulnerability to negative rumination and reinforcing the experience of loneliness (Malon et al., 2024). Moreover, it can induce doubts about how one is perceived by others, influencing self-disclosure and self-presentation in social contexts (Lemay et al., 2024).

Taken together, the self appears critical for understanding the psychological and behavioral consequences of loneliness (Akin, 2010). Yet, most prior research has focused on non-cognitive aspects such as self-reflection and self-disclosure, while fewer studies have examined how loneliness influences self-referential cognitive processing, which may provide important insights into the mechanisms linking loneliness and psychological functioning.

### 1.2 Loneliness and the self-reference effect in memory

The SRE refers to the phenomenon in which self-related information is processed more deeply and organized more elaborately than non-self-related information, yielding a memory advantage (Rogers et al., 1977; Rosa et al., 2024). Thus, the SRE reflects a distinct depth and bias in self-related information processing. To date, only one study has directly examined the relationship between loneliness and the SRE. Using personality adjectives and manipulating self, friend, and celebrity as reference targets, Kokici et al. (2023) found that high-loneliness individuals exhibited a stronger self-reference advantage relative to friend-reference, and friend-reference memory became more similar to celebrity-reference memory. This suggests that high-loneliness individuals may focus more on themselves at the expense of psychological connection with others.

Most previous SRE studies rely on memory performance of self- versus other-related information to define the effect. However, research on related constructs such as anxiety and depression suggests that affective biases can influence intentional forgetting, particularly for negative information. For instance, individuals with depression tend to maintain irrelevant negative information in working memory longer than healthy controls (Wen et al., 2023). It remains unclear whether high-loneliness individuals exhibit similar forgetting biases, particularly under self-referential processing.

### 1.3 The present study

Directed forgetting provides a useful framework for examining the regulation of memory, as it requires individuals to intentionally suppress previously encoded information. Compared to standard memory paradigms, it reflects a more complex cognitive process involving inhibitory control and memory updating. Prior research has shown that directed forgetting is sensitive to mental health status; for example, individuals with depression often show impaired forgetting of negative or self-referential information, indicating reduced flexibility in cognitive control (e.g., Gotlib et al., 2004).

Given that loneliness is associated with heightened self-focus and altered social information processing, incorporating a directed forgetting paradigm allows us to examine whether lonely individuals show difficulties in regulating self-referential information. The rationale for this prediction is not merely that loneliness is associated with general cognitive rigidity, but that social disconnection may specifically affect the inhibitory control of self-schema-related information. Loneliness reflects perceived social disconnection and is thought to trigger a “self-preservation” mode characterized by hypervigilance toward social threat and intensified self-focused processing (Cacioppo and Hawkley, 2009; Cacioppo and Cacioppo, 2018).

Consistent with social-monitoring accounts of loneliness (Gardner et al., 2005), this heightened self-focus may increase the accessibility and resistance to inhibition of self-related representations, making them more difficult to disengage from or suppress. Consistent with this view, loneliness has been associated with stronger self-referential memory biases and greater cognitive distance between the self and close others (Kokici et al., 2023). Directed forgetting provides a useful way to test this mechanism because it requires individuals to update memory according to current goals and inhibit information that is no longer task-relevant. Prior research suggests that self-referential information is particularly resistant to directed forgetting and may require greater inhibitory control to suppress (Yang et al., 2013; Mao et al., 2017). Therefore, if loneliness increases the functional salience and resistance to inhibition of self-schema-related information, high-loneliness individuals should show reduced ability to intentionally forget self-referential items. Experiment 2 was designed to test this possibility by combining the self-reference paradigm with directed forgetting instructions.

The present research employed a self-description paradigm across two experiments to examine how high- and low-loneliness individuals process self-referential memory under both remember and directed-forgetting conditions. Experiment 1 investigated memory accuracy for self-, friend-, and stranger-referenced items to replicate and extend prior findings (Kokici et al., 2023). Experiment 2 combined the self-description paradigm with a directed-forgetting task, using recall of “to-be-remembered” and “to-be-forgotten” items to explore forgetting mechanisms from a self-referential perspective. Given evidence that negative affective tendencies bias memory toward negative material (Gotlib et al., 2004), positive and negative two-character Chinese personality adjectives were included to examine valence-specific effects.

Based on prior research, we proposed three hypotheses:

H1: Consistent with prior findings (Kokici et al., 2023), high-loneliness individuals will exhibit a stronger self-reference effect (SRE) than low-loneliness individuals.

H2: Under directed-forgetting conditions, high-loneliness individuals will show reduced ability to intentionally forget self-referential items, maintaining stronger SREs even when such information is designated as to-be-forgotten.

H3: High-loneliness individuals will demonstrate a mnemonic advantage for negative words.

## 2 Experiment 1: Loneliness and the self-reference effect in a “remember” task

### 2.1 Method

#### 2.1.1 Participants

Sample size was determined using G*Power 3.1 (*α* = 0.05, *f* = 0.25, 1-*β* = 0.80), indicating a minimum of 34 participants (17 per group). A total of 124 undergraduate and graduate students completed the UCLA Loneliness Scale, from which 55 participants were selected. The high-loneliness group included 28 participants (21 females; Score range 42–64, *M* = 49.71, *SD* = 6.85), and the low-loneliness group included 27 participants (21 females; Score range 23–31, *M* = 27.48, *SD* = 2.41). All participants were right-handed, had normal or corrected-to-normal vision, and reported no history of mental illness.

#### 2.1.2 Materials

Loneliness was assessed using the UCLA Loneliness Scale Version 2 (Russell et al., 1978), consisting of 20 items rated on a 1–4 scale, with ten reverse-scored items. Higher scores indicate greater loneliness (Cronbach’s α = 0.91).

A total of 180 Chinese two-character personality adjectives were pre-rated by five graduate students for emotional valence and familiarity. Ninety were positive (*M*_valence_ = 4.70, *SD* = 0.29) and ninety negative (*M*_valence_ = 1.26, *SD* = 0.22). 120 adjectives were used as study items and 60 as distractors in the recognition test.

#### 2.1.3 Design

A 2 (Loneliness: high vs. low) × 2 (Valence: positive vs. negative) mixed design was employed. Loneliness served as the between-subjects factor, valence was treated as the within-subjects factor. To examine the self-reference effect, memory performance (item recognition accuracy and source memory accuracy) was computed separately for each reference condition (self, friend, stranger).

#### 2.1.4 Procedures

Prior to the formal task, participants nominated a close friend and provided impression ratings of general impressions for each reference target (self, friend, and stranger). These ratings were collected to characterize participants’ subjective perceptions of the targets. The mean ratings were as follows: *M*_Self_ = 7.16, *SD* = 1.30; *M*_friend_ = 7.87, *SD* = 0.98; *M*_Stranger_ = 7.82, *SD* = 0.96. These ratings were included for descriptive purposes only and were not used as a formal manipulation check.

The experiment consisted of three phases:

##### 2.1.4.1 Learning phase

Participants performed a self-referential judgment and memorization task. Each trial began with a fixation cross “+” displayed at the center of the screen for 500 ms, followed by the presentation of a two-character word. Participants were instructed to memorize the word while simultaneously judging whether the descriptor was most applicable to themselves, a close friend, or a stranger (the experimenter). Responses were made within a 3 s window by pressing the “1,” “2,” or “3” keys, respectively.

To control for item-specific effects, the assignment of personality adjectives to the three reference conditions (self, friend, and stranger) was fully counterbalanced across participants. Additionally, the presentation order of the items was randomized for each participant. Because the reference category was determined by participants’ own judgments, participants were instructed to make careful and balanced judgments across the three reference categories and to avoid overusing any single category.

To assess potential allocation bias, we calculated the proportion of words assigned to each reference category for each participant. These proportions were defined as “reference levels” and were used as baseline indices of encoding allocation (see Results).

##### 2.1.4.2 Distractor phase

Participants engaged in a 2-min mental arithmetic task. This involved performing serial subtractions (starting from 1,000 and subtracting 3 continuously) and recording their answers on a blank sheet of paper.

##### 2.1.4.3 Retrieval phase

The final phase consisted of an item recognition task followed by a source memory judgment task. A total of 180 words—120 previously studied (“old”) and 60 novel lures—were presented in a randomized order.

##### 2.1.4.4 Recognition

Participants had 3 s to indicate whether the word was “new” (pressing the F key) or “old” (pressing the J key).

##### 2.1.4.5 Source attribution

**Figure 1:**
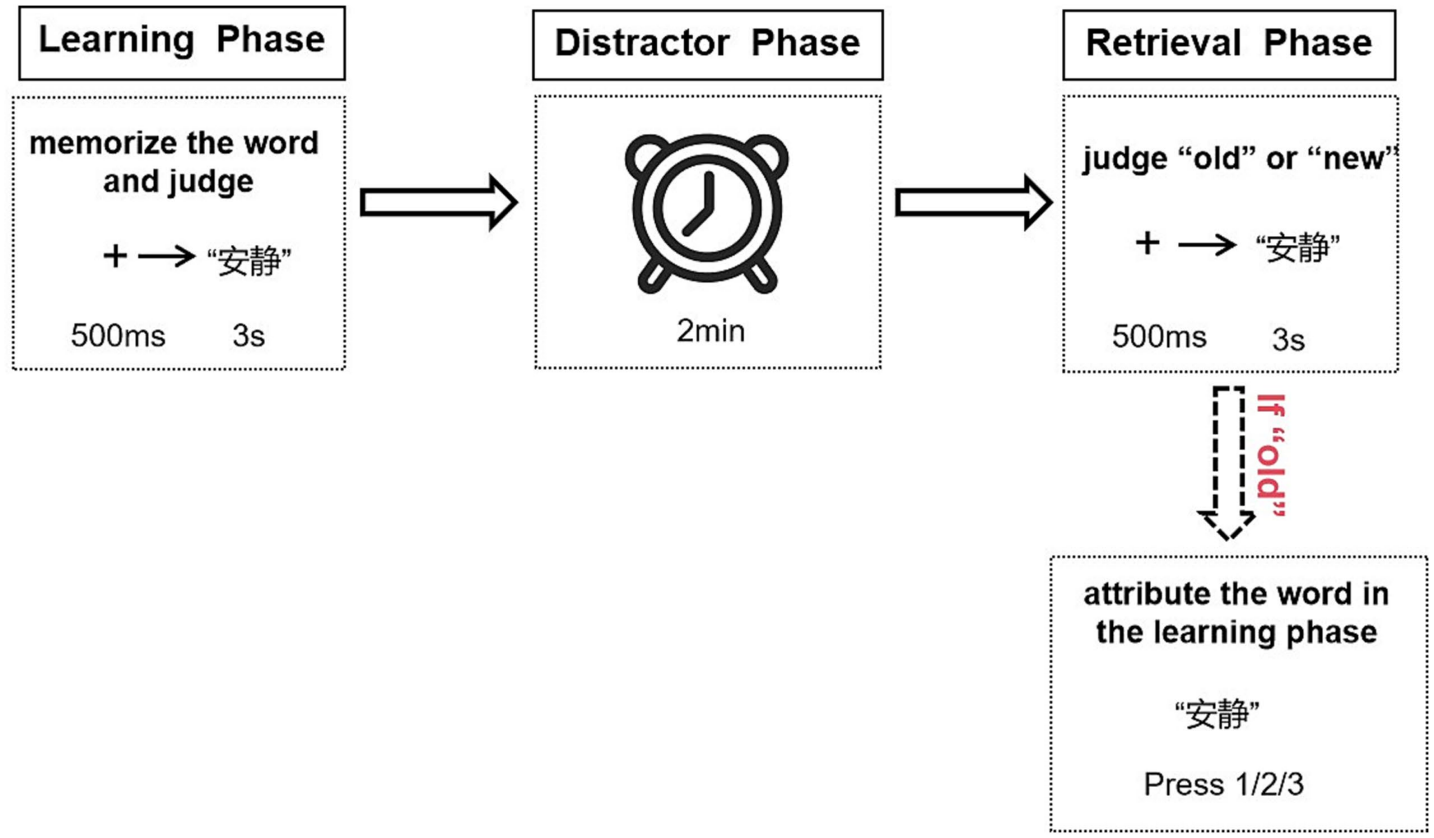
Experimental procedure flowchart. The experiment comprised three phases: (1) Learning phase with self-reference judgments; (2) distractor phase (mental arithmetic); and (3) retrieval phase including recognition and source memory tasks.

If a word was identified as “old,” a follow-up prompt appeared: “To whom did you attribute this word during the learning phase?” Participants identified the original category by pressing “1” (self), “2” (friend), or “3” (stranger) (see Figure 1).

### 2.2 Results

#### 2.2.1 Recognition accuracy by valence

**Figure 2:**
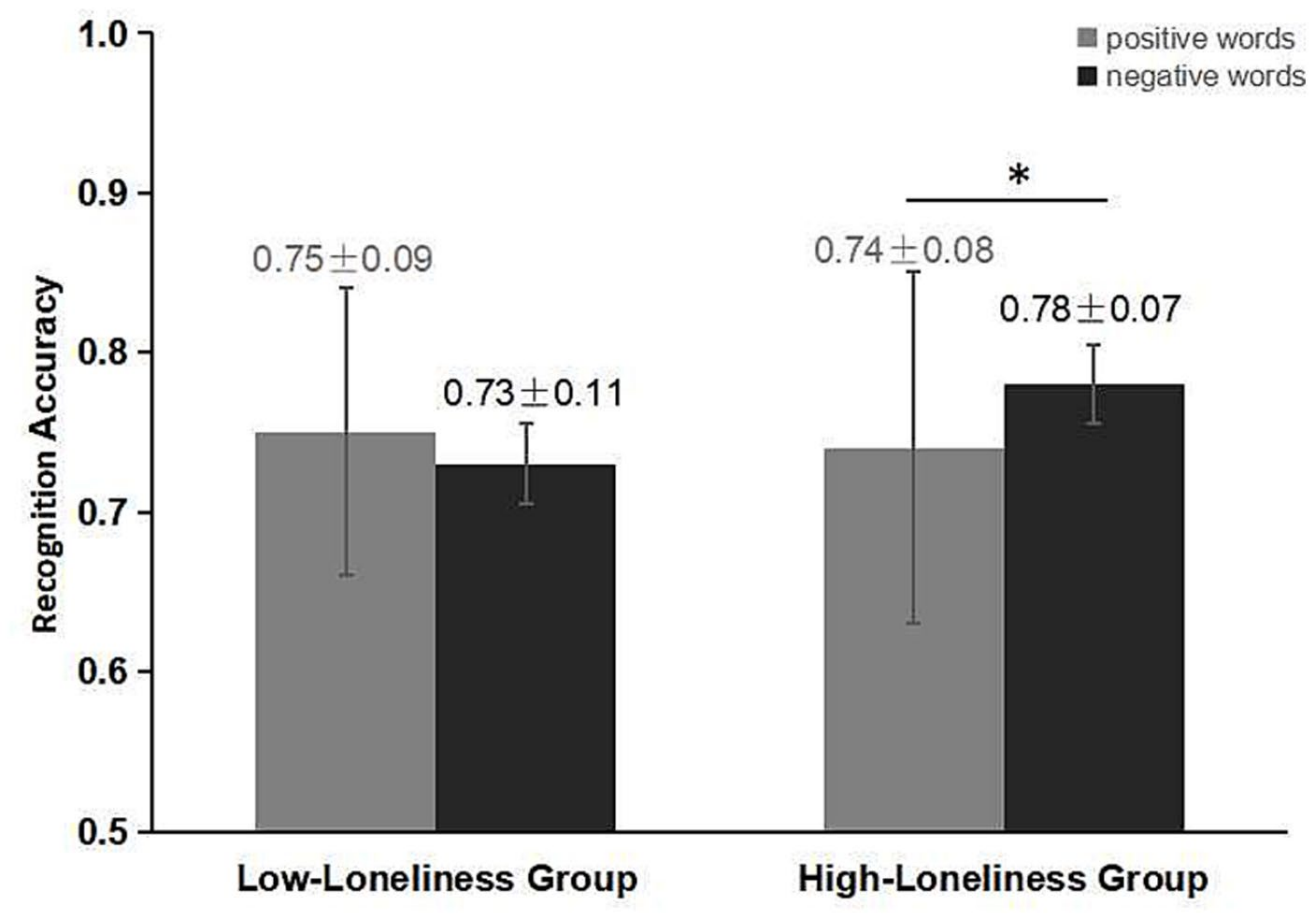
Mean recognition accuracy as a function of loneliness group (high vs. low) and word valence (positive vs. negative) in experiment 1.

A 2 (loneliness level: high vs. low) × 2 (word valence: positive vs. negative) repeated-measures ANOVA was performed on recognition accuracy. The results revealed no significant main effects for either loneliness level, *F*(1, 53) = 0.92, *p* > 0.05, or word valence, *F*(1, 53) = 2.48, *p* > 0.05. However, the interaction between loneliness level and word valence was significant, *F*(1, 53) = 7.45, *p* < 0.01, η_p_^2^ = 0.12. Simple effects showed that the high-loneliness group recognized negative words more accurately than positive words (*p* < 0.05), whereas the low-loneliness group showed no valence difference (*p* > 0.05) (see Figure 2).

#### 2.2.2 Reference levels

To examine whether participants’ allocation of reference categories was balanced, we calculated the proportion of words assigned to each reference category during encoding for each participant. These proportions, referred to as “reference levels,” served as baseline indices of allocation tendency rather than memory performance per se.

Independent samples t-tests were conducted to compare these levels between the high- and low-loneliness groups. The analysis yielded no significant group differences for self-reference (*t* = −0.31, *p* = 0.76), friend-reference (*t* = −0.87, *p* = 0.39), or stranger-reference levels (*t* = −0.56, *p* = 0.58) (see Table 1).

**Table 1:** Three Reference Levels (*M* ± *SD*).

Reference level	Low-loneliness group	High-loneliness group
Self-reference	0.50 ± 0.13	0.49 ± 0.11
Friend-reference Stranger-reference	0.31 ± 0.08 0.19 ± 0.10	0.30 ± 0.09 0.20 ± 0.10

#### 2.2.3 Reference effects

**Figure 3:**
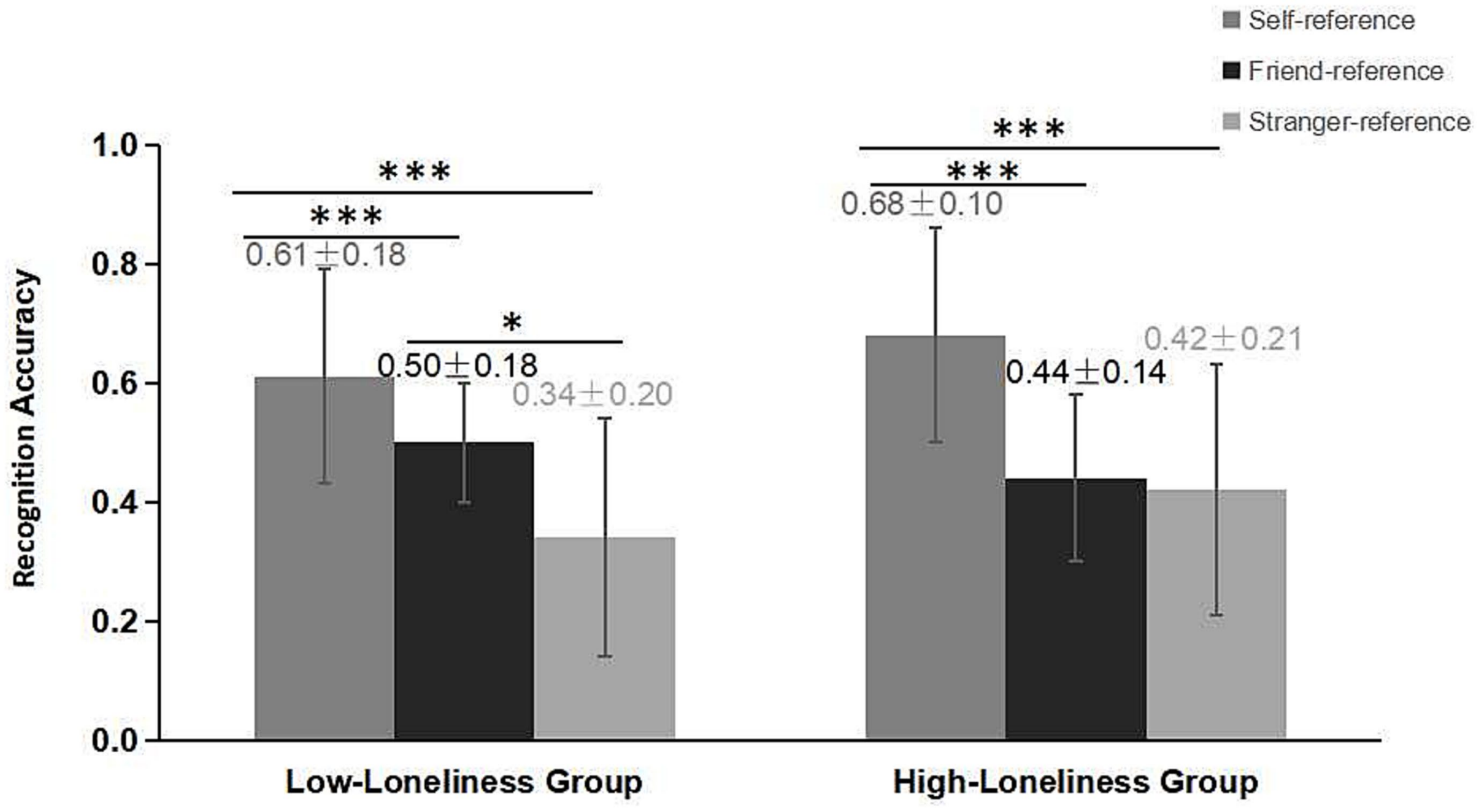
Recognition accuracy for self-, friend-, and stranger-referenced items across high- and low-loneliness groups in experiment 1.

To evaluate the reference effect, recognition accuracy for words associated with different targets was analyzed. A 2 (loneliness level: high vs. low) × 3 (reference target: self vs. friend vs. stranger) repeated-measures ANOVA was conducted on recognition accuracy. Results revealed a significant main effect of reference target, *F*(2, 52) = 42.92, *p* < 0.001, η_p_^2^ = 0.45. Post-hoc comparisons indicated a clear hierarchy in memory performance: self-reference accuracy [*M*_self_ = 0.65, *SD* = 0.02, 95% *CI* = (0.61, 0.69)] was significantly superior to both friend-reference [*M*_friend_ = 0.47, *SD* = 0.02, 95% *CI* = (0.42, 0.51)] and stranger-reference accuracy [*M*_stranger_ = 0.38, *SD* = 0.03, 95% *CI* = (0.33, 0.44)], with friend-reference also significantly exceeding stranger-reference (*ps* < 0.05). The main effect of loneliness level was not significant, *F*(1, 53) = 0.69, *p* > 0.05. However, the interaction between loneliness and reference target was significant, *F*(2, 52) = 3.76, *p* < 0.05, η_p_^2^ = 0.07. Simple effects analysis showed that while the self-reference advantage persisted in both groups (*ps* < 0.001), the distinction between social targets varied by loneliness level. Specifically, participants in the low-loneliness group exhibited significantly higher accuracy for friend-references than for stranger-references (*p* < 0.05). In contrast, this “friend-stranger” distinction vanished in the high-loneliness group (*p* > 0.05), suggesting a diminished differentiation between close and distant social targets (see Figure 3).

### 2.3 Interim discussion

Experiment 1 demonstrated a robust SRE: recognition accuracy for self-referential words was significantly higher than for both friend- and stranger-referential words, with friend-reference also exceeding stranger-reference. These findings replicate the well-established mnemonic advantage of self-referential processing (Brédart, 2016), confirming that information encoded in relation to the self is processed more deeply and retrieved more efficiently than information associated with others.

Importantly, loneliness modulated the reference effect. In the low-loneliness group, participants showed a clear differentiation between friend- and stranger-references, indicating intact processing of interpersonal relationship information. In contrast, the high-loneliness group exhibited no significant difference between friend- and stranger-references, suggesting that elevated loneliness narrows the distinction between close and distant social targets. This pattern aligns with previous research indicating that loneliness may disrupt the encoding and retrieval of social information, particularly regarding close others, potentially due to reduced interpersonal engagement and weaker social connectedness (Kokici et al., 2023; Bruss et al., 2024).

Additionally, a valence-specific memory bias emerged in the high-loneliness group: negative words were recognized more accurately than positive words, whereas no such valence effect was observed in the low-loneliness group. This finding is consistent with the Mood-Congruence Hypothesis (Blaney, 1986) and prior evidence that socially and emotionally isolated individuals preferentially attend to and retain negative information (Cacioppo and Patrick, 2008). The absence of a valence bias in the low-loneliness group may reflect more effective emotion regulation and a balanced attentional focus toward positive and negative information (Gross, 2002).

Taken together, Experiment 1 suggests that loneliness influences both the SRE and valence-specific memory processing. Specifically, high loneliness appears to amplify self-focused attention, diminish differentiation between social targets, and bias memory toward negative information. These effects likely reflect multiple cognitive mechanisms, including attentional bias, memory encoding, and retrieval processes, as well as socio-emotional influences on cognitive functioning.

An open question remains: do these effects persist when individuals are required to intentionally modulate memory, as in a directed forgetting task? Experiment 2 was designed to address this question by examining how high- and low-loneliness individuals perform under “remember” versus “forget” instructions, providing insight into the interplay between loneliness, self-referential processing, and cognitive flexibility.

## 3 Experiment 2: The self-reference effect of loneliness on memory in a directed forgetting task

### 3.1 Method

#### 3.1.1 Participants

The required sample size was determined using the same parameters as Experiment 1 (*α* = 0.05, *f* = 0.25, 1-*β* = 0.80), confirming a minimum of 34 participants. A total of 59 undergraduate and graduate students were selected from 146 respondents based on their UCLA Loneliness Scale scores. Participants’ ages ranged from 18 to 25 years (*M* = 19.68, *SD* = 1.33). The high-loneliness group included 31 participants (26 females; Score range 42–65, *M* = 49.87, *SD* = 5.67), and the low-loneliness group included 28 participants (23 females; Score range 21–30, *M* = 26.73, *SD* = 2.75). All participants were right-handed, had normal or corrected-to-normal vision, and no color blindness or color weakness.

#### 3.1.2 Materials

Stimuli were identical to those employed in Experiment 1.

#### 3.1.3 Design

A 2 (Loneliness: high vs. low) × 2 (Valence: positive vs. negative) mixed design was employed. Loneliness was treated the between-subjects factor, valence was treated the within-subjects factor. In addition, memory instruction (remember vs. forget) was manipulated within participants. To investigate memory regulation, the directed forgetting effect, defined as the difference in performance between “remember” and “forget” conditions, was used as the primary dependent measure. This directed forgetting effect was examined across the three reference conditions (self, friend, stranger).

#### 3.1.4 Procedure

Prior to the formal task, participants nominated a close friend and provided impression ratings of general impressions for each reference target (self, friend, and stranger). These ratings were collected to characterize participants’ subjective perceptions of the targets. The mean ratings were as follows: *M*_self_ = 6.64, *SD* = 1.11; *M*_friend_ = 7.76, *SD* = 1.04; *M*_stranger_ = 7.80, *SD* = 0.89. These ratings were not used as a formal manipulation check, but served only as descriptive measures.

The experimental procedure closely followed that of Experiment 1, with the key modification that the learning phase included a directed forgetting task. Specifically, participants first judged whether a word described themselves, a close friend, or the experimenter by pressing “1,” “2,” or “3.” Immediately after, a “remember” or “forget” instruction was presented for 500 ms, and participants were instructed to engage in internal cognitive operations according to the instruction (e.g., rehearsing the word for “remember” or suppressing it for “forget”). The assignment of “remember” and “forget” instructions to adjectives was fully counterbalanced across participants and presented in a randomized order within each participant. Because items were presented randomly, the probability that a given instruction (remember vs. forget) co-occurred with each reference category (self, friend, or stranger) was statistically equivalent across the experiment. The retrieval phase was identical to Experiment 1, consisting of an old/new recognition task followed by a source memory judgment for words identified as “old.”

### 3.2 Results

#### 3.2.1 Recognition accuracy by word valence

**Figure 4:**
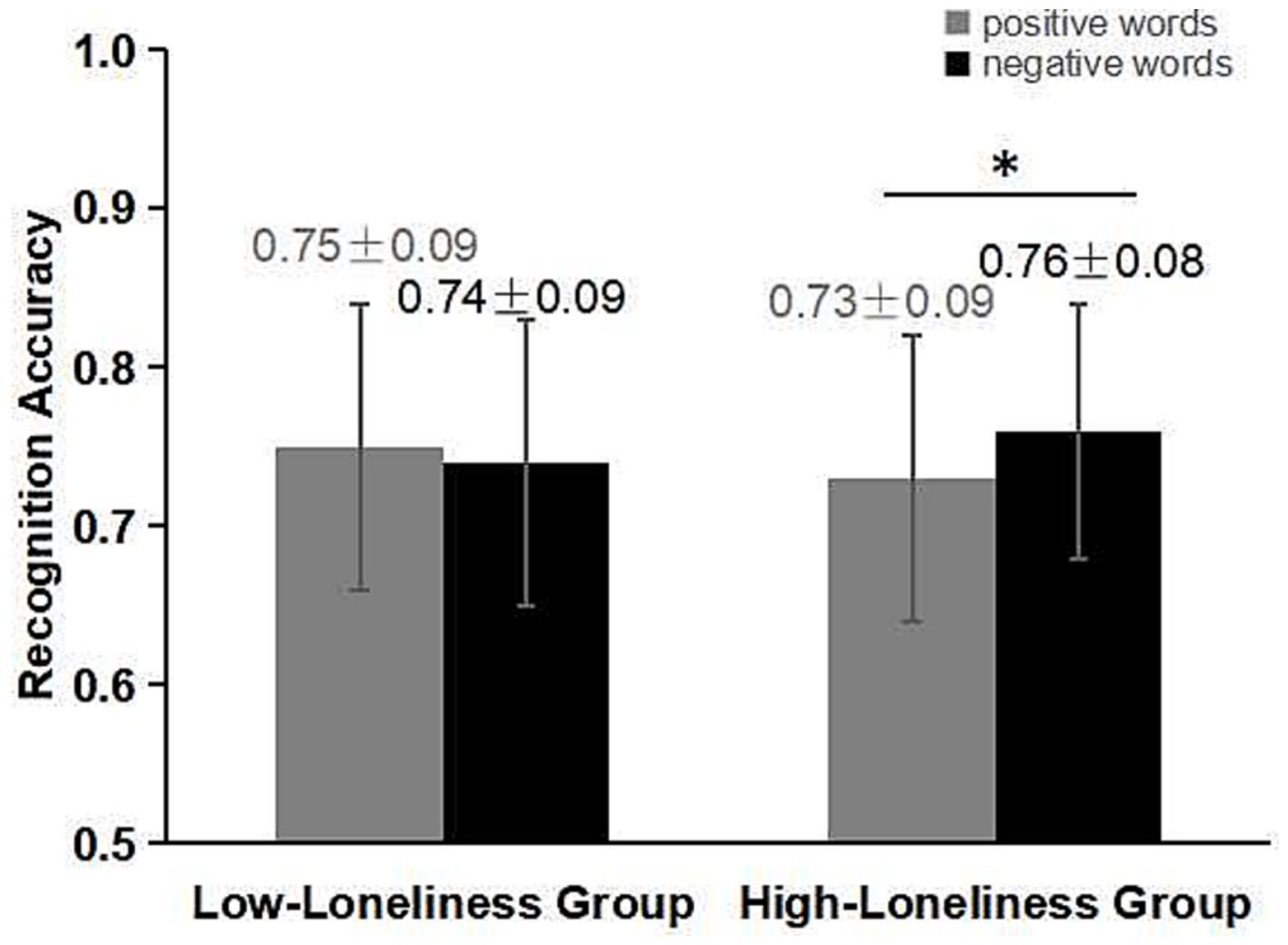
Interaction between loneliness group and word valence on recognition accuracy in experiment 2.

Ignoring memory instructions, a 2 (loneliness: high vs. low) × 2 (word valence: positive vs. negative) repeated-measures ANOVA on recognition accuracy revealed no significant main effects of loneliness, *F*(1, 57) = 0.02, *p* > 0.05, or word valence, *F*(1, 57) = 1.44, *p* > 0.05. The interaction between loneliness and valence was significant, *F*(1, 57) = 5.46, *p* < 0.05, η_p_^2^ = 0.09. Simple effects analysis indicated that high-loneliness participants recognized negative words more accurately than positive words (*p* < 0.05), whereas low-loneliness participants showed no significant valence difference (*p* > 0.05) (see Figure 4).

#### 3.2.2 Reference levels

As in Experiment 1, reference levels were calculated as the proportion of words assigned to each reference category during encoding for each participant. These values served as baseline indices of allocation tendency and were used to assess whether allocation differed across groups. Independent samples t-tests revealed no significant differences between high- and low-loneliness groups for self-reference (*t* = 0.27, *p* = 0.79), friend-reference (*t* = 0.41, *p* = 0.69), or stranger-reference (*t* = −0.64, *p* = 0.53) levels (see Table 2).

**Table 2:** Three Reference Levels (*M* ± *SD*).

Reference level	Low-loneliness group	High-loneliness group
Self-reference	0.44 ± 0.10	0.45 ± 0.09
Friend-reference	0.30 ± 0.08	0.31 ± 0.05
Stranger-reference	0.26 ± 0.11	0.24 ± 0.08

#### 3.2.3 Reference effect

**Figure 5:**
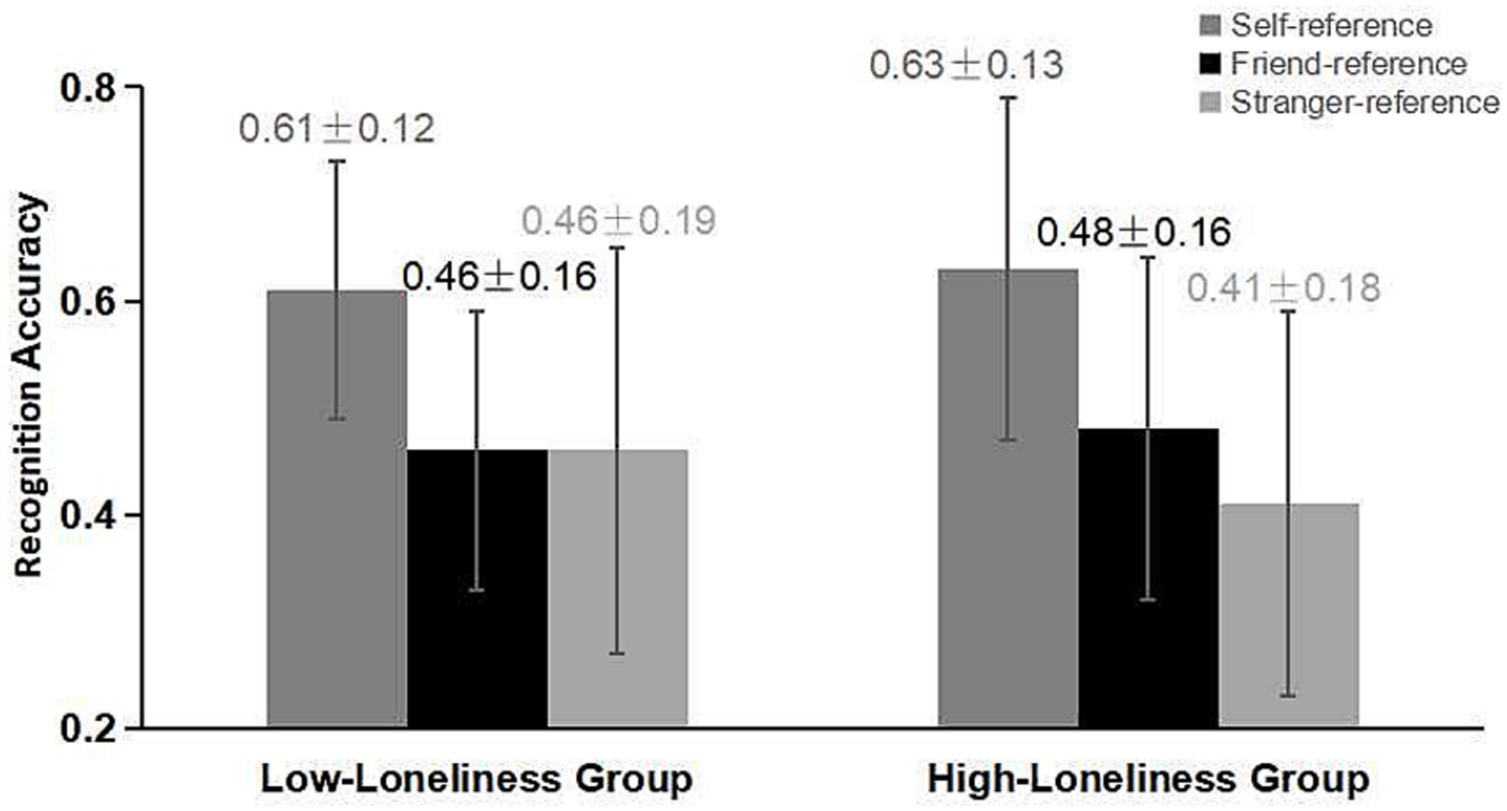
Overall recognition accuracy for the three reference targets (self, friend, stranger) in Experiment 2, collapsed across memory instructions.

Ignoring the “remember” or “forget” instructions, a 2(loneliness level: high vs. low) × 3 (reference target: self vs. friend vs. stranger) repeated-measures ANOVA on recognition accuracy revealed a significant main effect of reference target, *F*(2, 56) = 49.28, *p* < 0.001, η_p_^2^ = 0.46. Recognition accuracy followed a clear hierarchy: self-reference > friend-reference > stranger-reference [*M*_self_ = 0.62, *SD* = 0.02, 95% *CI* = (0.59, 0.65); *M*_friend_ = 0.47, *SD* = 0.02, 95% *CI* = (0.43, 0.51); *M*_stranger_ = 0.44, *SD* = 0.02, 95% *CI* = (0.39, 0.49)]. Neither the main effect of loneliness, *F*(1, 57) = 0.00, *p* > 0.05, η_p_^2^ < 0.001, nor the interaction between loneliness and reference target, *F*(2, 56) = 2.27, *p* > 0.05, η_p_^2^ = 0.04, was significant (see Figure 5).

#### 3.2.4 Recognition accuracy under “remember” and “forget” instructions

To directly examine the joint effects of loneliness level and memory instruction on reference-related memory performance, a 2 (loneliness level: high vs. low) × 2 (memory operation: remember vs. forget) × 3 (reference target: self vs. friend vs. stranger) mixed-design ANOVA was conducted on recognition accuracy.

The main effect of loneliness level was not significant, *F*(1, 57) = 0.00, *p* > 0.05, η_p_^2^ = 0.00. However, the main effect of memory instruction was significant, *F*(1, 57) = 5.15, *p* < 0.05, η_p_^2^ = 0.08, indicating slightly higher recognition accuracy in the “remember” condition [*M*_remember_ = 0.261, *SD* = 0.07, 95% *CI* = (0.24, 0.28)] than in the “forget” condition [*M*_forget_ = 0.247, *SD* = 0.07, 95% *CI* = (0.23, 0.27)]. The main effect of reference target was also significant, *F*(2, 56) = 47.21, *p* < 0.001, η_p_^2^ = 0.45, *Post hoc* tests revealed that self-referential items were recognized more accurately [*M*_self_ = 0.31, *SD* = 0.01, 95% *CI* = (0.30, 0.33)] than both friend-referential [*M*_friend_ = 0.23, *SD* = 0.01, 95% *CI* = (0.21, 0.25)] and stranger-referential items [*M*_stranger_ = 0.22, *SD* = 0.01, 95% *CI* = (0.20, 0.24)], which did not differ significantly from each other.

Critically, the three-way interaction among loneliness level, memory instruction, and reference target was significant, *F*(2, 56) = 5.51, *p* < 0.01, η_p_^2^ = 0.09. This interaction directly addresses the combined effects of loneliness level and memory instruction, indicating that the influence of memory instruction on reference-related memory performance differs across loneliness groups.

To further interpret this interaction, follow-up analyses examined the effect of reference target within each instruction condition for both groups.

**Figure 6:**
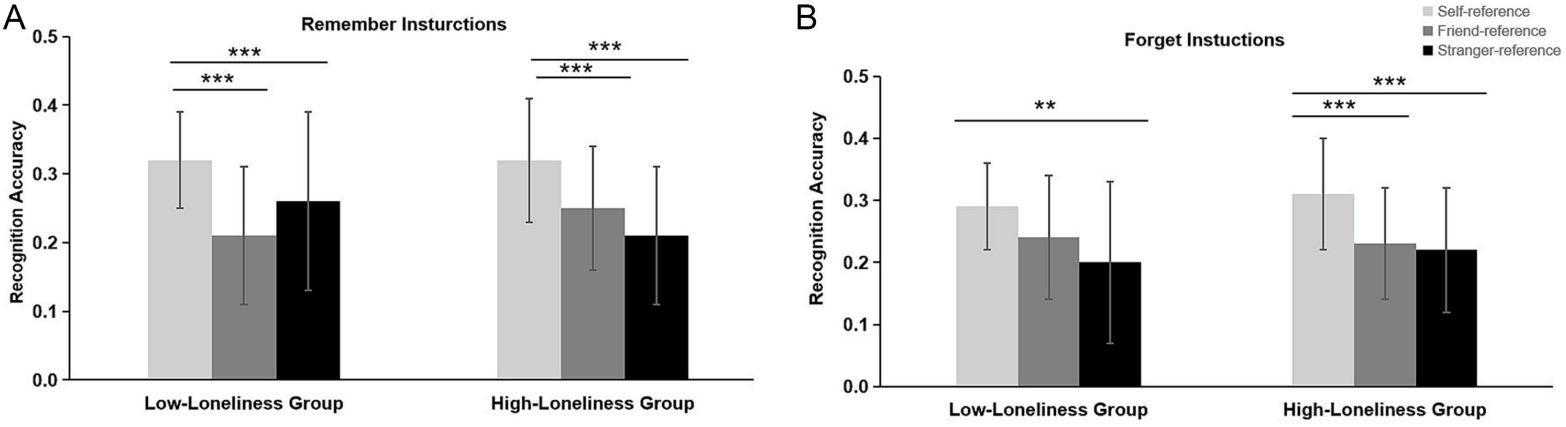
Recognition accuracy for reference targets as a function of loneliness group under “remember” **(A)** and “forget” **(B)** instructions.

Under the “remember” instruction, both the high-loneliness [*F*(2, 60) = 15.55, *p* < 0.001] and low-loneliness groups [*F*(2, 54) = 8.61, *p* < 0.001] showed a clear self-reference advantage. In the high-loneliness group, self-referential items were recognized more accurately than both friend- and stranger-referential items [*M*_self_ = 0.32, *SD* = 0.09, 95% *CI* = (0.29, 0.36); *M*_friend_ = 0.25, *SD* = 0.09, 95% *CI* = (0.22, 0.29); *M*_stranger_ = 0.21, *SD* = 0.10, 95% *CI* = (0.16, 0.23)]. Similarly, in the low-loneliness group, self-referential accuracy was higher than other categories, whereas friend- and stranger-referential items did not differ significantly [*M*_self_ = 0.32, *SD* = 0.07, 95% *CI* = (0.30, 0.35); *M*_friend_ = 0.21, *SD* = 0.10, 95% *CI* = (0.17, 0.25); *M*_stranger_ = 0.26, *SD* = 0.13, 95% *CI* = (0.21, 0.31)] (see Figure 6 Left Panel).

Under the “forget” instruction, a different pattern emerged between the two groups. In the high-loneliness group, the self-reference advantage remained significant, *F*(2, 60) = 8.67, *p* < 0.001 with self-referential items continuing to be recognized more accurately than other targets [*M*_self_ = 0.31, *SD* = 0.07, 95% CI (0.28, 0.33); *M*_friend_ = 0.23, *SD* = 0.09, 95% *CI* = (0.20, 0.26); *M*_stranger_ = 0.22, *SD* = 0.11, 95% *CI* = (0.18, 0.26)]. In contrast, in the low-loneliness group, although the overall effect of Reference Target remained significant, *F*(2, 54) = 5.90, *p* < 0.01. *Post hoc* analyses showed that self-referential items were recognized more accurately than stranger-referential items, whereas recognition accuracy for friend-referential items did not differ significantly from either self- or stranger-referential items [*M*_self_ = 0.29, *SD* = 0.08, 95% *CI* = (0.25, 0.32); *M*_friend_ = 0.24, *SD* = 0.09, 95% *CI* = (0.20, 0.27); *M*_stranger_ = 0.20, *SD* = 0.11, 95% *CI* = (0.16, 0.24)] (see Figure 6 Right Panel).

### 3.3 Interim discussion

In the context of the directed forgetting task, Experiment 2 replicated the core findings of Experiment 1 by demonstrating a robust self-reference advantage over both friend- and stranger-referential processing. Regardless of memory instruction, self-referential items were consistently recognized more accurately than other-referential items, indicating that the SRE remained stable even when participants were required to intentionally regulate memory.

Consistent with Experiment 1, individuals with high loneliness again exhibited a mnemonic bias toward negative words, whereas no valence-related difference was observed in the low-loneliness group. This pattern suggests that the preferential retrieval of negative information among high-loneliness individuals is not task-specific, but rather reflects a stable memory bias that persists across both simple remembering and directed forgetting contexts.

More importantly, Experiment 2 revealed group differences in how memory instructions modulated reference-related processing. Under the remember instruction, both high- and low-loneliness groups showed a similar reference pattern: in addition to a strong self-reference advantage, recognition accuracy for friend- and stranger-referential items did not differ. This pattern suggests that explicit remembering instructions effectively enhanced encoding or retrieval of non-self-referential information, particularly diminishing social-distance-related differences in the low-loneliness group.

Under the forget instruction, however, the two groups diverged. The high-loneliness group continued to exhibit the same rigid reference pattern observed under the remember instruction, characterized by a stable self-reference advantage and no differentiation between friend and stranger references. In contrast, the low-loneliness group showed a relative reduction in self-referential recognition accuracy, resulting in a weakened self-reference advantage. This finding suggests that low-loneliness individuals were more capable of suppressing self-referential information when instructed to forget, whereas high-loneliness individuals were less responsive to such top-down memory control demands.

Taken together, these findings indicate that low-loneliness individuals possess greater flexibility in implementing internal memory operations in accordance with task instructions, effectively enhancing or suppressing self-referential processing as required. In contrast, high loneliness appears to be associated with a more rigid self-referential processing style, in which self-related information remains highly accessible regardless of intentional attempts to forget.

## 4 General discussion

The present study examined how loneliness influences self-referential memory and its regulation under directed forgetting instructions. Consistent with previous research, a robust SRE was observed across both experiments. Importantly, the present findings extend prior work by demonstrating that loneliness not only shapes what information is preferentially remembered, but also modulates how flexibly self-referential information can be regulated under conditions requiring cognitive control. Specifically, while both high- and low-loneliness groups exhibited a self-reference advantage under remember instructions, only the low-loneliness group showed a reduction of this advantage under forget instructions, whereas the high-loneliness group maintained it.

### 4.1 Loneliness modulates recognition bias for emotional valence

Across both experiments, loneliness reliably moderated the relationship between emotional valence and recognition performance. Individuals with low loneliness showed comparable recognition accuracy for positive and negative words, suggesting a relatively balanced encoding and retention of emotionally valenced information. In contrast, individuals with high loneliness consistently exhibited a recognition advantage for negative words.

This pattern aligns with prior evidence indicating that loneliness is associated with negative cognitive biases in social and emotional information processing (Cacioppo and Hawkley, 2009; Sviarkaltsava, 2016). According to the social monitoring system framework, loneliness heightens vigilance toward potential social threats, leading individuals to preferentially attend to and process negative social cues. Supporting this account, Cacioppo, Balogh et al. (2015) demonstrated that high-loneliness individuals show greater Stroop interference when processing negative social stimuli, reflecting enhanced implicit processing of negative information.

From an emotional memory perspective, loneliness has also been linked to asymmetric recall patterns, characterized by diminished retrieval of positive social experiences but preserved or even enhanced recall of negative ones (Igarashi, 2025). Together, these findings suggest that individuals with high loneliness do not merely attend more to negative information, but process it more deeply and retain it more effectively. In the present study, this tendency likely reflects negative expectations about both the self and others, which bias encoding and consolidation toward negative material (Bellucci, 2020). Thus, loneliness appears to shape recognition performance not by globally impairing memory, but by systematically biasing the prioritization and consolidation of emotionally negative information.

### 4.2 Loneliness and referential memory structure across remembering and forgetting

Although loneliness did not alter the absolute advantage of self-reference, it substantially affected how non-self social targets were differentiated in memory. In Experiment 1, which involved intentional remembering only, the low-loneliness group exhibited a clear hierarchical structure of referential memory (self > friend > stranger), consistent with objective differences in interpersonal closeness. In contrast, the high-loneliness group showed no significant distinction between friend- and stranger-referential items.

This convergence of friend- and stranger-reference in the high-loneliness group suggests that loneliness narrows the cognitive representation of social relationships. On the one hand, heightened self-focus may reduce attentional resources allocated to others; on the other hand, limited social interaction and weakened intimate bonds may lead to less differentiated representations of close versus distant social targets. This interpretation is consistent with prior findings that loneliness attenuates psychological connectedness with close others, rendering friend-related processing more similar to that of strangers (Kokici et al., 2023). These findings suggest that loneliness may fundamentally alter the cognitive architecture of social representation. Rather than maintaining a finely graded representation of interpersonal closeness, high-loneliness individuals appear to rely on a simplified distinction between self and others. This reduction in representational differentiation may reflect both decreased social engagement and a shift toward self-focused processing, which together limit the encoding of nuanced interpersonal information.

Importantly, this interpretation does not imply that low-loneliness individuals maintain a rigidly hierarchical social memory structure across all task contexts. Rather, the pattern across experiments suggests that their referential memory structure is task-sensitive. Whereas the low-loneliness group showed a clear friend–stranger distinction in Experiment 1, this distinction was attenuated under the explicit “remember” instruction in Experiment 2, suggesting that intentional memory goals can temporarily reduce the influence of interpersonal distance.

### 4.3 Directed forgetting and cognitive flexibility in loneliness

From a cognitive control perspective, directed forgetting paradigms are widely interpreted as reflecting the efficiency of inhibitory control and memory updating processes. Experiment 2 further revealed that loneliness modulates how flexibly individuals implement intentional memory control. Regardless of whether items were designated to be remembered or forgotten, the high-loneliness group consistently exhibited the same referential pattern observed in Experiment 1: a strong self-reference advantage accompanied by an undifferentiated “friend–stranger” category. This invariance across task demands suggests a rigid self-referential processing style that is relatively insensitive to top-down memory instructions.

In contrast, the low-loneliness group demonstrated marked task-dependent modulation of referential memory. Under the “remember” instruction, these individuals also showed a flattened friend–stranger pattern, suggesting that intentional memory goals can temporarily reduce the influence of interpersonal distance. Rather than reflecting impaired social differentiation, this pattern may indicate goal-directed cognitive flexibility. For individuals with low loneliness, who may possess relatively stable social representations and are less characterized by interpersonal hypervigilance, explicit remembering may allow task demands to override habitual relational biases, leading to a more balanced allocation of memory resources across social targets. This differs qualitatively from the flattened friend–stranger pattern observed in high-loneliness individuals, which appeared more stable across task contexts and may reflect structural blurring of social schemas. Under the “forget” instruction, self-referential processing was effectively suppressed, resulting in a weakened self-reference advantage. These shifts indicate that low-loneliness individuals are better able to flexibly allocate cognitive resources in accordance with task goals.

Directed forgetting is widely understood to rely on selective rehearsal and inhibitory control mechanisms, which require efficient cognitive switching and flexibility (Tan et al., 2020). Across the three task conditions examined in the present study—intentional remembering, to-be-remembered items, and to-be-forgotten items—the high-loneliness group consistently showed a two-level referential structure (“self” vs. “friend–stranger”). This pattern likely reflects not only increased self-focus and social disengagement but also reduced cognitive flexibility, a characteristic previously associated with loneliness (Wang et al., 2020).

By contrast, the low-loneliness group exhibited a dynamic reconfiguration of referential memory structure across task contexts, reflecting greater adaptability in internal memory operations. Notably, this enhanced flexibility may also contribute to the absence of valence bias in this group, as flexible cognitive control supports balanced processing of emotional information.

Importantly, these findings have potential practical implications. If loneliness is associated with reduced flexibility in regulating self-referential information, this may contribute to persistent self-focused cognitive patterns such as rumination. Interventions targeting cognitive control processes may therefore be beneficial. Such approaches may be particularly relevant in clinical or educational settings, where reducing maladaptive self-focused processing could improve emotional well-being and social functioning.

### 4.4 Limitations and future directions

Several limitations of the present study should be acknowledged. First, the sample consisted primarily of young adults drawn from a non-clinical population. Although this sampling approach is common in experimental research on self-referential processing and memory, it remains unclear whether the observed patterns would generalize to older adults or individuals experiencing chronic or clinically significant loneliness. Future studies could extend the present findings by examining more diverse age groups and clinical populations to clarify the boundary conditions of loneliness-related effects on self-referential memory.

Second, the present study relied exclusively on behavioral measures of memory performance, such as recognition accuracy and signal detection indices. While the findings are consistent with interpretations in terms of altered cognitive flexibility and inhibitory control under loneliness, these underlying mechanisms were not directly assessed. Future research combining behavioral paradigms with neurocognitive approaches, including electrophysiological or neuroimaging methods, may provide more direct evidence regarding the processes that support intentional control and referential differentiation in memory. Future studies could further examine whether experimentally modifying cognitive control processes or self-referential biases can causally influence loneliness-related outcomes.

Third, in terms of stimuli selection, the present study used personality adjectives, which may not fully reflect memory processes in real-world social interactions. Prior research suggests that images, due to their rich visual features, are generally more memorable than words (Schurgin, 2018; Goujon et al., 2022; Yin et al., 2024), as they capture attention more effectively and facilitate deeper encoding. In contrast, the abstract nature of adjectives may limit the emotional and sensory richness of self-referential processing. Nevertheless, the use of verbal materials allows for precise experimental control and helps isolate the cognitive mechanisms underlying self-referential memory and its regulation. Future studies could improve ecological validity by using more naturalistic and multi-modal materials, such as faces or social scenes, to test whether the observed effects generalize to more realistic contexts.

Finally, we note that using the experimenter as the “other” referent may introduce contextual features (e.g., physical presence) which could influence encoding processes and should be further examined in future research, for example by comparing different types of “other” referents.

## 5 Conclusion

The present study leads to three primary conclusions. First, individuals with high loneliness exhibit a reliable memory advantage for negative words, whereas individuals with low loneliness show balanced memory performance across emotional valence. Second, loneliness influences referential memory structure: high-loneliness individuals consistently fail to differentiate between friend- and stranger-referenced information across remembering and forgetting contexts, while low-loneliness individuals show flexible, task-dependent reference patterns. Third, these effects likely stem from core cognitive features of loneliness, including heightened self-focus, reduced social differentiation, and diminished cognitive flexibility.

By integrating self-reference and directed forgetting paradigms, this study provides novel evidence that loneliness affects not only what individuals remember, but also how flexibly they can regulate self-referential memory. These findings offer important insights into the cognitive mechanisms through which loneliness may sustain negative biases and impair adaptive social functioning.

## Data Availability

The raw data supporting the conclusions of this article will be made available by the authors, without undue reservation.
